# A Novel Apoptosis-Related Gene Signature Predicts Biochemical Recurrence of Localized Prostate Cancer After Radical Prostatectomy

**DOI:** 10.3389/fgene.2020.586376

**Published:** 2020-11-30

**Authors:** Qijie Zhang, Kai Zhao, Lebin Song, Chengjian Ji, Rong Cong, Jiaochen Luan, Xiang Zhou, Jiadong Xia, Ninghong Song

**Affiliations:** ^1^Department of Urology, The First Affiliated Hospital of Nanjing Medical University, Nanjing, China; ^2^Department of Dermatology, The First Affiliated Hospital of Nanjing Medical University, Nanjing, China; ^3^The Affiliated Kezhou People's Hospital of Nanjing Medical University, Xinjiang, China

**Keywords:** apoptosis-related gene signature, prostate cancer, radical prostatectomy, biochemical recurrence, prognosis

## Abstract

**Background:** Nowadays, predictions of biochemical recurrence (BCR) in localized prostate cancer (PCa) patients after radical prostatectomy (RP) are mainly based on clinical parameters with a low predictive accuracy. Given the critical role of apoptosis in PCa occurrence and progression, we aimed to establish a novel predictive model based on apoptosis-related gene signature and clinicopathological parameters that can improve risk stratification for BCR and assist in clinical decision-making.

**Methods:** Expression data and corresponding clinical information were obtained from four public cohorts, one from The Cancer Genome Atlas (TCGA) dataset and three from the Gene Expression Omnibus (GEO) dataset. Weighted gene co-expression network analysis (WGCNA) was performed to identify candidate modules closely correlated to BCR, and univariate and multivariate Cox regression analyses were utilized to build the gene signature. Time-dependent receiver operating curve (ROC) and Kaplan–Meier (KM) survival analysis were used to assess the prognostic value. Finally, we analyzed the expression of genes in the signature and validated the results using quantitative real-time PCR (qRT-PCR).

**Results:** The novel gene signature we established exhibited a high prognostic value and was able to act as an independent risk factor for BCR [Training set: *P* < 0.001, hazard ratio (HR) = 7.826; Validation set I: *P* = 0.006, HR = 2.655; Validation set II: *P* = 0.003, HR = 4.175; Validation set III: *P* < 0.001, HR = 3.008]. Nomogram based on the gene signature and clinical parameters was capable of distinguishing high-risk BCR patients. Additionally, functional enrichment analysis showed several enriched pathways and biological processes, which might help reveal the underlying mechanism. The expression results of qRT-PCR were consistent with TCGA results.

**Conclusion:** The apoptosis-related gene signature could serve as a powerful predictor and risk factor for BCR in localized PCa patients after RP.

## Introduction

Prostate cancer (PCa) remains the second most frequently diagnosed malignancy in men worldwide (Siegel et al., 2020). According to the statistics of the American Cancer Society, there will be 191,930 newly diagnosed PCa cases and about 33,330 men died of PCa in America in 2020. Due to advances in monitoring methods and medical examinations, many patients have been diagnosed with PCa at an early stage. For these clinically localized PCa patients, evidence-based guidelines recommend radical prostatectomy (RP) as the primary treatment and most of them will benefit from this procedure (Attard et al., 2016; Wallis et al., 2016; Ilic et al., 2017). However, it has been reported that ~15–40% of patients will develop biochemical recurrence (BCR) after RP and finally progress to castration-resistant PCa (Ghadjar et al., 2018; Teo et al., 2019). For these patients, more active follow-up and personalized adjuvant therapy, such as androgen deprivation therapy, chemotherapy, and radiation therapy, should be taken to improve prognosis. To avoid overtreatment, it is necessary and meaningful to accurately identify patients with a high risk of BCR.

Previous studies revealed that Gleason score (GS), prostate-specific antigen (PSA), surgical margin (SM), and clinical T stage (cT) were selected as parameters for risk stratification for BCR after local therapy (Mottet et al., 2017). Nowadays, the availability of large-scale public databases with gene expression data and clinical data makes it possible to construct a more accurate prognostic signature than conventional clinical parameters. These molecular biomarkers could provide not only additional prognostic information but also insight into the mechanisms of BCR in PCa. Apoptosis, also known as programmed cell death, is involved in several biological and pathological processes, such as embryonic development, homeostatic maintenance of tissues and organs, oncogenesis, and tumor progression (Majno and Joris, 1995; Tang and Porter, 1996). Additionally, alternations in apoptosis pathways play an important role in resistance to conventional antitumor therapies, such as radiotherapy, chemotherapy, and targeted therapy (Lim et al., 2019). In PCa, two major apoptotic pathways, the death receptor-mediated pathways (extrinsic pathway) and the mitochondria-mediated pathway (intrinsic pathway), are involved in the process of tumor progression and recurrence (Hirata et al., 2009; Khan et al., 2014). As we know, there is no existing apoptosis-related gene signature for predicting BCR in localized PCa patients after RP.

Considering the important role of apoptosis in the progression and recurrence of PCa, we aimed to establish a novel apoptosis-related prediction model to improve risk stratification of BCR in localized PCa patients after RP. These results might contribute to a better understanding of the underlying mechanism of BCR, including the role of apoptosis.

## Materials and Methods

### Publicly Available Datasets and Apoptosis-Related Genes

All relevant datasets were identified by comprehensively searching NCBI Gene Expression Omnibus (GEO) datasets before May 10, 2020. The search strategy consisted of the following keywords: “*Homo sapiens*” and “Series” and “Expression profiling by array” and “prostate cancer” and (“recurrence” or “recurrent”). Expression data along with all available clinical information were retrieved for PCa patients from The Cancer Genome Atlas (TCGA). The inclusion criteria were as follows: (1) biospecimens were collected from patients with localized PCa undergoing RP, (2) containing at least 50 samples in each dataset, and (3) including both clinical parameters (PSA, cT, SM, or GS) and outcomes (BCR).

After screening, four independent cohorts were eventually included in our research, containing a total of 688 PCa samples along with corresponding clinical information. Among them, one cohort was from TCGA database and three cohorts were from the GEO database. The RNA-seq data of 379 patients were accessed from TCGA, and RNA-seq data of 106 patients from GSE54460 were produced with the Affymetrix Human Exon 1.0 ST Array. Additionally, the microarray data of 92 patients from GSE70769 and 111 patients from GSE70768 were from the same research (Ross-Adams et al., 2015), which were produced with the same chip platform (Illumina HumanHT-12 V4.0 Array). Probe IDs were mapped to gene symbols based on the relevant annotation information, and expression measurements of all probes linking to the same gene symbol were averaged to obtain a single value. The list of apoptosis-related genes was extracted from the Molecular Signature Database v7.1 (https://www.gsea-msigdb.org/gsea/msigdb/index.jsp). The search strategy consisted of the following keywords: “apoptosis” or “apoptosis” and “*Homo sapiens*.” Eventually, 181 gene sets containing 2,411 unique genes were included in the list (Supplementary Material 1). TCGA cohort was taken as a training set, while the other three cohorts were used for validation. All RNA-seq data and microarray data were normalized and log2 transformed with the manufacturer-provided R packages.

### Candidate Selection and Signature Construction

The expression data of 2,411 apoptosis-related genes in the training set were used to construct a scale-free co-expression network with the weighted gene co-expression network analysis (WGCNA) R package (Langfelder and Horvath, 2008). First, hierarchical clustering analysis of PCa patients with different clinical characteristics (BCR, GS, and cT) was performed, based on the expression of apoptosis-related genes, to detect outliers. Next, the fit soft threshold power (β) was screened to ensure the construction of scale-free networks based on the Pearson's correlation coefficient between apoptosis-related genes. In this study, β = 3 (details in Supplementary Material) was selected to build a scale-free network. The topological overlap matrix (TOM) was constructed based on the adjacency, and the corresponding dissimilarity (1-TOM) was used as the distance measure, with a minModuleSize of 30, to assign apoptosis-related genes into different modules *via* hierarchical clustering analysis. Unassigned genes were categorized into a gray module. Then, we identified the modules that were significantly correlated with the clinical traits based on two parameters, module eigengenes and gene significance. Among non-gray modules, the modules with the highest absolute correlations with BCR were chosen as candidate modules for further analysis. Univariate Cox regression analysis was performed to screen for prognostic apoptosis-related genes in the candidate module, and the latter were enrolled in the multivariate Cox regression analysis to establish the prognostic risk model. The risk score was calculated as follows:

$$\begin{array}{l} \text{Riskscore}=\overset{n}{\underset{i=1}{\sum }}expi^{*}\beta i \end{array}$$

where n is the number of prognostic genes, expi is the expression level of prognostic gene i, and βi is the regression coefficient of gene i.

### Functional Enrichment Analysis

Gene Ontology (GO) annotation and Kyoto Encyclopedia of Genes and Genomes (KEGG) pathway analysis of the genes in the candidate module were conducted using the Database for Annotation, Visualization, and Integrated Discovery (DAVID) online tool (version 6.8; https://david.ncifcrf.gov/). Moreover, gene set enrichment analysis (GSEA) software, which was downloaded from Broad Institute (http://www.broadinstitute.org/gsea/index.jsp), was used to analyze the potential pathways underlying the gene signature with gene set “hallmark.all.v7.1.symbols.gmt” based on RNA-seq data in the training set.

### Construction of the Nomogram

To assist clinical procedures and improve risk stratification, a nomogram model (Iasonos et al., 2008), which integrated the gene signature and prognostic clinicopathological features, was built as a quantitative tool to predict BCR in PCa patients. The calibration plot and time-dependent receiver operating characteristics (ROC) analysis were used to investigate the calibration and discrimination of the model.

### Cell Culture

The human PCa cell line (LNCaP) and normal myofibroblast stromal cell line (WPMY-1) were obtained from the Institute of Biochemistry and Cell Biology of the Chinese Academy of Sciences (Shanghai, China). The cells were cultured in Roswell Park Memorial Institute 1640 or Dulbecco's Modified Eagle's Medium (Gibco BRL, Carlsbad, CA, USA) supplemented with 10% heat-inactivated fetal bovine serum (Gibco BRL), 100 U/ml penicillin sodium, and 100 mg/ml streptomycin sulfate at 37°C in a humidified air atmosphere with 5% CO_2_.

### RNA Isolation and Quantitative Real-Time PCR

Total RNA was extracted from cell lines using TRIzol reagent (Invitrogen Life Technologies, Carlsbad, CA, USA) according to the manufacturer's instructions. The isolated RNA was reverse transcribed into cDNA using a reverse transcription kit (Vazyme, Nanjing, China). According to the manufacturer's protocols, reverse transcription was conducted at 37°C for 15 min, followed by 85°C for 5 s. Quantitative real-time PCR (qRT-PCR) was performed using a standard protocol from SYBR Green Mix (Vazyme) to detect the expression of the candidate genes. Each 10 μl of the PCR reaction volume comprised SYBR Premix (2×, 5 μl), forward primer (10 μM, 0.2 μl), reverse primer (10 μM, 0.2 μl), cDNA sample (1 μl), and bidistilled water (3.6 μl). The qRT-PCR reaction was performed on an ABI StepOnePlus instrument (Applied Biosystems, Carlsbad, CA, USA). The relative expression of each mRNA was calculated and normalized by the 2^−ΔΔCt^ method relative to β-actin. Each experiment was performed in triplicate. The primer sequences used in this study were listed in Table 1.

**Table 1:** Quantitative real time PCR primers.

**Primer**	**Primer sequence (5**^****′****^**-3**^****′****^**)**	
**Name**	**Forward**	**Reverse**
NLRP12	ACCAGACCTTGACCGACCTT	GAGGACTCGGAGTTTGCAGC
CDKN2A	ATGGAGCCTTCGGCTGACT	GTAACTATTCGGTGCGTTGGG
STX4	CTGTCCCAGCAATTCGTGGAG	CCCAGCATTGGTGATCTTCAG
RAB27A	GGAGAGGTTTCGTAGCTTAACG	CCACACAGCACTATATCTGGGT
HSF1	GCACATTCCATGCCCAAGTAT	GGCCTCTCGTCTATGCTCC
AURKB	CAGAAGAGCTGCACATTTGACG	CCTTGAGCCCTAAGAGCAGATTT
BTG-2	CCTGTGGGTGGACCCCTAT	GGCCTCCTCGTACAAGACG
PHLDA3	ACATCTACTTCACGCTGGTG	CTGCTGGTTCTTGAACTTGAC
E2F1	ATAGTGTCACCACCACCATCAT	GAAAGGCTGATGAACTCCTCAG
NSMF	CGAGCGTTTGGAGAGTACCTG	TGCGGGCTTCCTAATGCTG
MSX1	GAAGATGCGCTCGTCAAAG	CTTACGGTTCGTCTTGTGTTTG
TPT1	GAAAGCACAGTAATCACTGGTGT	ACGGTAGTCCAATAGAGCAACC
ERP29	AAGAGAGCTACCCAGTCTTCTA	TTCTTCTGAGTCTCCTTCACAC
MT1F	TGCGCCGCTGGTGTCT	GACGCCCCTTTGCAAACA
ADGRB1	ATGACCGACTTCGAGAAGGACG	TCTGCGGCATCTGGTCAATGTG
β-actin	CCACCATGTACCCAGGCATT	CGGACTCATCGTACTCCTGC

### Statistics

Levene's test and Kolmogorov–Smirnov test were used to assess the normality and homogeneity of variance. For parametric variables, Student's t-test or one-way analysis of variance (ANOVA) was used for continuous variables and the chi-square test or Fisher exact test for categorical variables. The Mann–Whitney and Wilcoxon tests were used for nonparametric variables. Survival curves were generated and analyzed by the Kaplan–Meier method using the log-rank test. The Cox proportional hazards regression model was applied to assess the prognostic value of each parameter for BCR. Time-dependent ROC analysis was used to measure the predictive power with the “survivalROC” R packages, and the areas under the ROC curve (AUC) of each variable at different time nodes were compared. Meta-analysis (*I*^2^ <50%, fixed-effect model) was performed to evaluate the prognostic value in the pooled cohort. The *Z*-score method was used to normalize the risk scores in each cohort. All statistical analyses were performed using IBM SPSS Statistics 24.0 and R software 3.6.3. A two-tailed *P* < 0.05 was considered to be statistically significant for all statistical analyses.

## Results

### Construction of an Apoptosis-Related Gene Signature for Biochemical Recurrence

**Figure 1:**
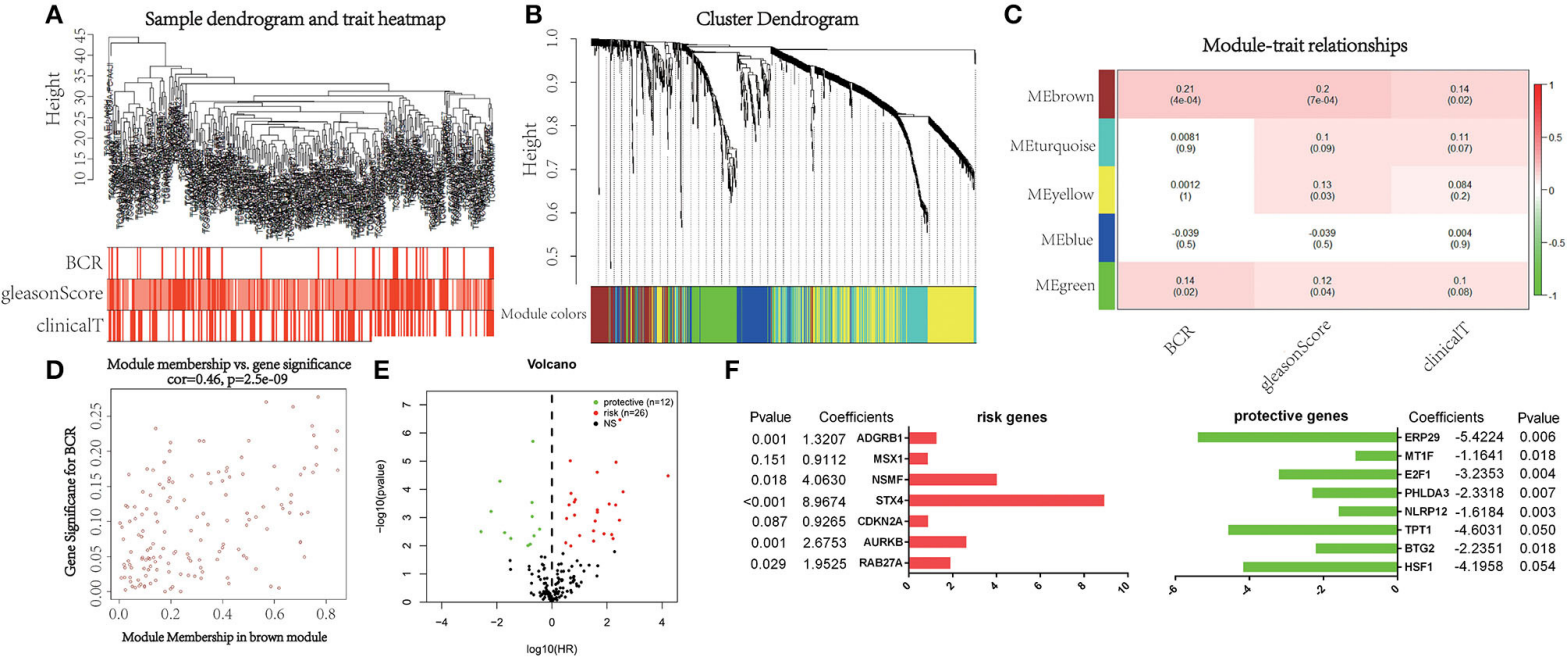
Selection of powerful biomarkers to construct a prognostic apoptosis-related gene signature. **(A)** Cluster tree of prostate cancer (PCa) samples in The Cancer Genome Atlas (TCGA) and the color band under the tree indicating the numeric values of clinical traits. **(B)** Cluster Dendrogram indicating different apoptosis-related gene modules. **(C)** Heatmap showing the correlation between the modules and clinical traits. **(D)** Scatterplot of gene significance for biochemical recurrence (BCR) vs. module membership in the brown module. **(E)** Volcano plot displaying the result of univariate Cox regression analysis. **(F)** Distribution of regression coefficients of the gene signature.

WGCNA was performed with RNA-seq data and clinical traits (BCR, GS, and cT) on the training set (Figure 1A). Sample clustering was performed to exclude outliers (Supplementary Figure 1). A total of five non-gray modules were obtained through a one-step network construction method, where β = 3 (Figure 1B). Then, we performed a correlation analysis between these non-gray modules and clinical traits. The results showed that among the non-gray modules, the brown module had the most significant correlation with not only BCR but also GS and cT (Figure 1C). Additionally, the distribution of the modules' average gene significance related to BCR was displayed in Figure 1D and Supplementary Material, among which the brown module also had the strongest correlation with BCR. Therefore, 152 genes from this module were selected for further univariate Cox regression analysis. With the threshold of *P* < 0.01, it turned out that 38 genes (12 protective and 26 risk genes) were significantly associated with BCR (Figure 1E). Next, multivariate Cox regression analysis was applied to develop a gene signature based on 38 prognostic genes. Finally, 15 genes (*RAB27A, HSF1, BTG2, AURKB, TPT1, NLRP12, PHLDA3, CDKN2A, STX4, E2F1, NSMF, MSX1, ADGRB1, MT1F*, and *ERP29*) were used to construct the gene signature. The distribution of regression coefficients of the gene signature was shown in Figure 1F.

### Predictive Value of Gene Signature for Biochemical Recurrence

**Figure 2:**
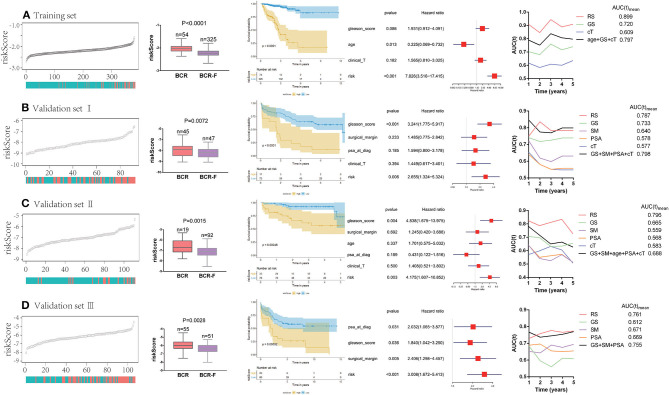
Predictive value of gene signature for biochemical recurrence (BCR) in each cohort **(A–D)**. Risk scores of patients with BCR were obviously higher than the ones without BCR. Patients with a high risk had a significantly poorer recurrence-free survival (RFS) than those with a low risk. Multivariate Cox regression analysis demonstrated that risk score was an independent prognostic factor for BCR. Time-dependent receiver operating curve (ROC) analysis showed that risk score also acted as a powerful predictor of BCR.

We ranked the risk scores of all patients in the training set, and the risk scores of patients with BCR were obviously higher than those of patients without BCR (*P* < 0.0001). Patients with high risk had a significantly poorer recurrence-free survival (RFS) than those with low risk (*P* < 0.0001). Multivariate Cox regression analysis demonstrated that the risk score was an independent prognostic factor for BCR [hazard ratio (HR) = 7.826, *P* < 0.001]. Additionally, time-dependent ROC analysis showed that the risk score also acted as a powerful predictor of BCR, with an average AUC of 0.899 after 5 years of follow-up (Figure 2A).

To further verify the reliability of the results, the same analyses were conducted in the other three independent validation sets (Figures 2B–D). It turned out that patients with BCR had significantly higher risk scores than those without BCR in all validation sets (validation I: *P* = 0.0072; validation II: *P* = 0.015; validation III: *P* = 0.0028). The Kaplan–Meier analysis in the three validation sets showed that low-risk scores were closely associated with better RFS (validation I: *P* < 0.0001; validation II: *P* = 0.0005; validation III: *P* = 0.0003). Notably, the risk score was always found to be an independent risk factor for BCR in all validation sets (validation I: HR = 2.655, *P* = 0.006; validation II: HR = 4.175, *P* = 0.003; validation III: HR = 3.008, *P* < 0.001), which was consistent with the result from the training set. Moreover, the powerful predictive value of the risk score was observed in the three validation sets, with an average AUC of 0.787, 0.796, and 0.761, respectively. Compared with clinical parameters, the risk score had the largest AUC in both training set and validation sets. Moreover, compared with the combination of clinical variables (age, cT, GS, SM, and PSA), the average AUC of the risk score was larger in three of the databases (training set, validation II, and validation III).

**Figure 3:**
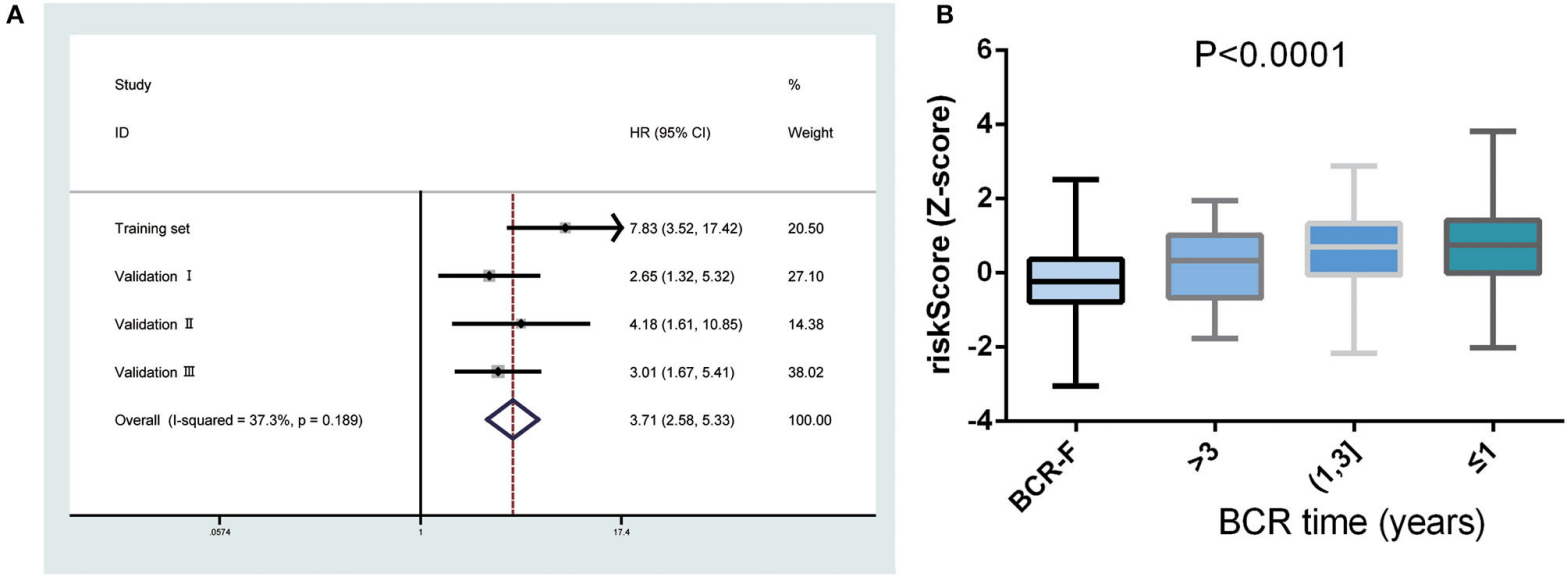
Predictive value of gene signature for biochemical recurrence (BCR) in the pooled cohort. **(A)** Meta-analysis showing that high-risk score yielded a worse recurrence-free survival (RFS). **(B)** *Z*-scores in BCR-free patients were significantly lower than those in BCR patients and had a tendency to increase gradually with the extension of BCR time.

Next, a meta-analysis was conducted to explore the prognostic value of the gene signature in the pooled cohort. The results showed that a high-risk score yielded a worse RFS in the pooled cohort (pooled HR = 3.71, 95% CI 2.58–5.33) (Figure 3A). We also calculated *Z*-scores to normalize risk scores and found that *Z*-scores in BCR-free patients were significantly lower than those in BCR patients. In addition, with the extension of BCR time, *Z*-scores tended to increase gradually (Figure 3B).

### Combination With Clinical Variables to Build a Predictive Nomogram

**Figure 4:**
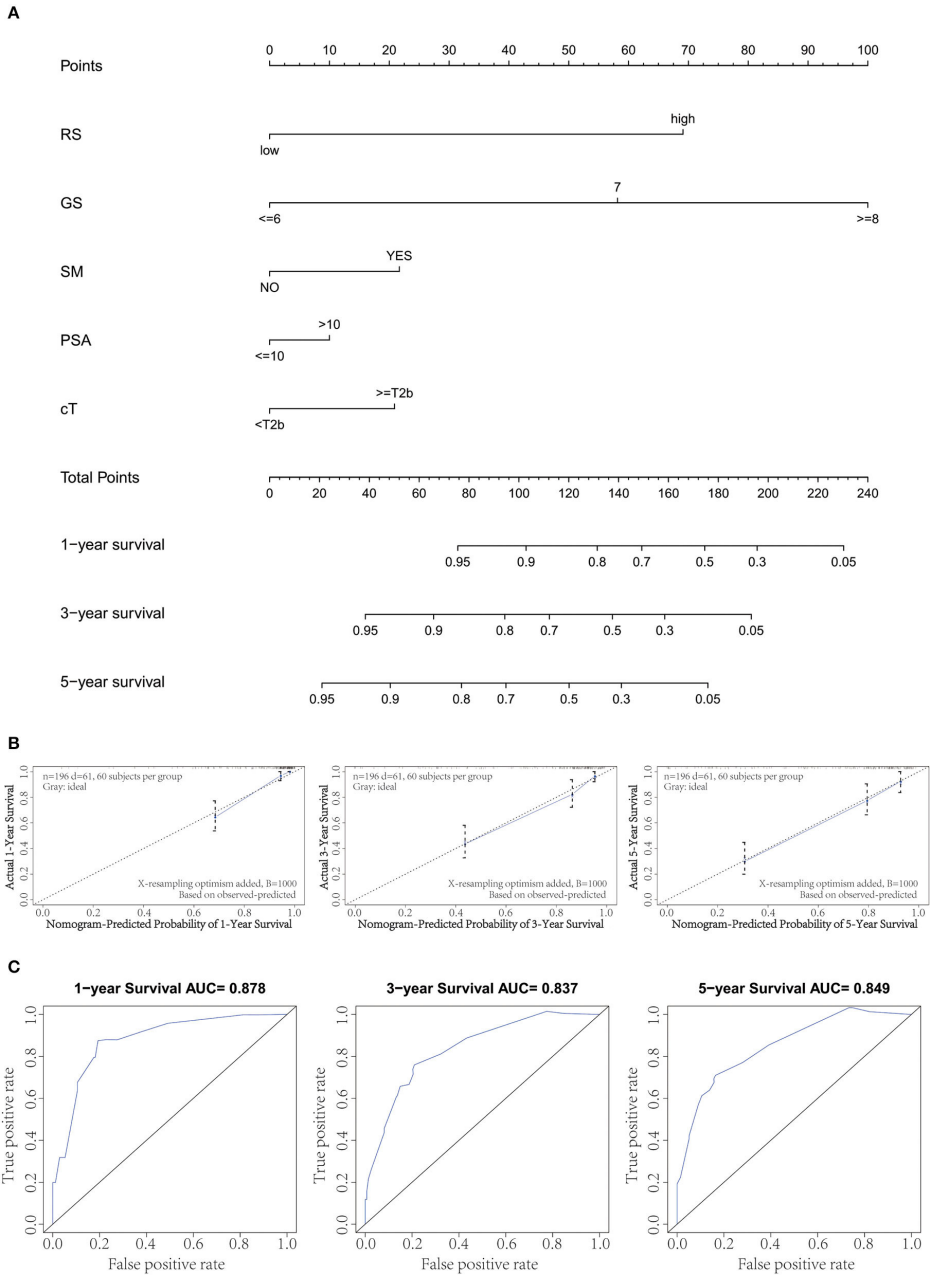
Combination of clinical variables to build a predictive nomogram. **(A)** Nomogram plot to predict 1-, 3-, and 5-year survival. **(B)** Calibration plot of the nomogram to predict 1-, 3-, and 5-year survival. **(C)** Receiver operating curve (ROC) curves of the nomogram to predict 1-, 3-, and 5-year survival.

By integrating the gene signature and four clinical parameters (GS, PSA, SM, and cT) in two cohorts (GSE70769 and GSE70768), we constructed a nomogram to meet the needs of clinicians to quantify the possible risk of BCR (Figure 4A). This allowed us to calculate the estimated possibility of BCR in PCa patients at 1, 3, and 5 years by plotting a vertical line between the total points and each prognosis axis. The AUC values of 1-, 3-, and 5-year nomograms were 0.878, 0.837, and 0.849, respectively (Figure 4B). Calibration curves of the nomogram showed no deviations from the reference line, and no recalibration was required (Figure 4C). Additionally, we established a nomogram based on clinical features and the AUC values of 1-year, 3-year, and 5-year BCR were 0.812, 0.733, and 0.748, respectively (Supplementary Figure 2).

### Functional Enrichment Analysis

**Figure 5:**
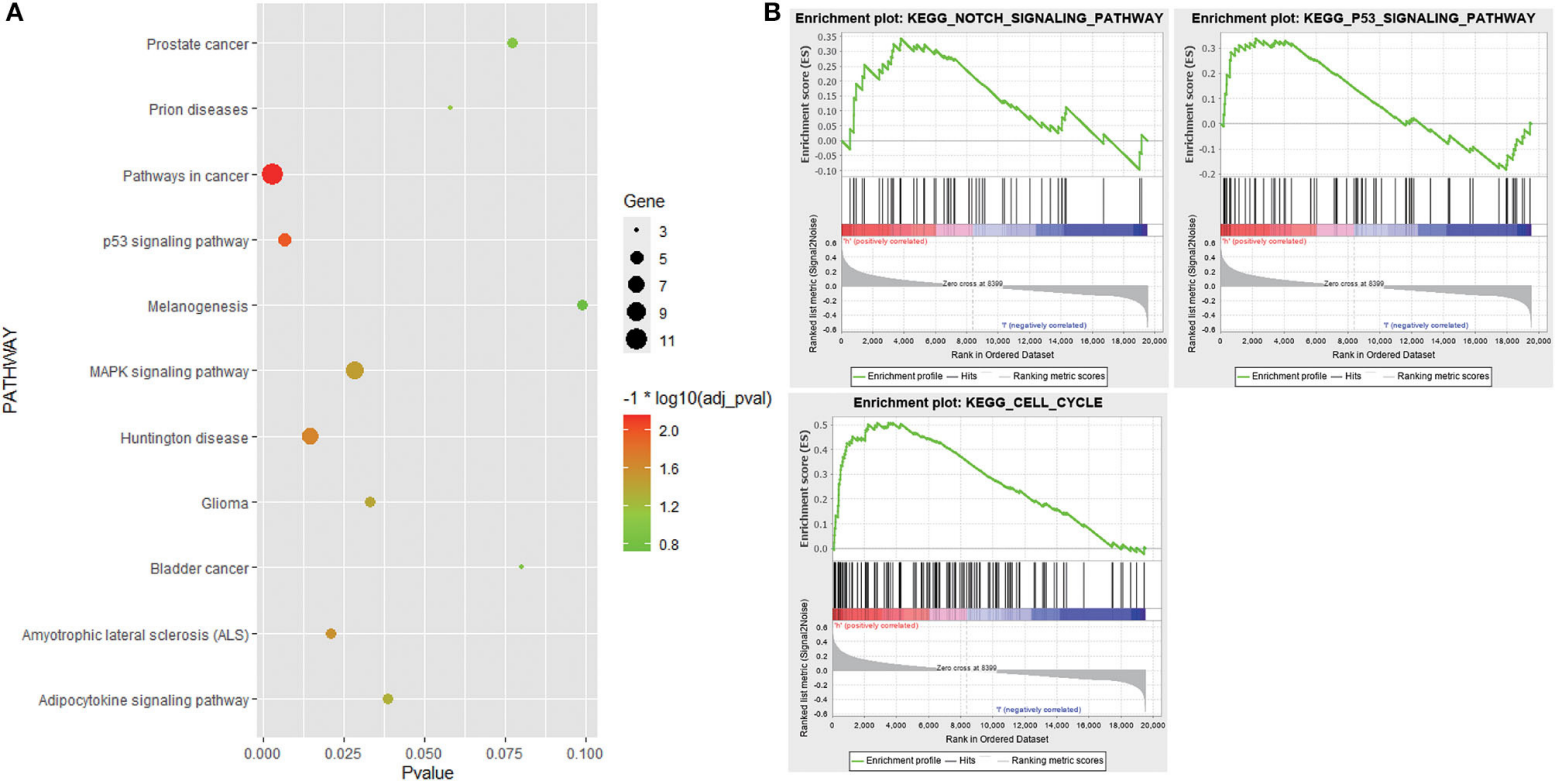
Functional enrichment analysis. **(A)** Bubble chart showing Kyoto Encyclopedia of Genes and Genomes (KEGG) pathways that 152 genes in the candidate module were enriched in. **(B)** KEGG pathways enriched in the high-risk group based on stratified gene set enrichment analysis (GSEA).

GO analysis results showed that 152 genes in the candidate module were significantly enriched in some apoptosis-related biological processes, such as regulation of apoptosis, regulation of programmed cell death, and regulation of cell death. As for the cell component, these genes were mainly involved in extracellular space, extracellular region part, and cytoplasmic membrane-bounded vesicle. In terms of molecular function, these genes were mainly related to identical protein binding, eukaryotic cell surface binding, and protein dimerization activity. KEGG pathway analysis revealed that these genes were associated with pathways in cancer, p53 signaling pathway, and mitogen-activated protein kinase (MAPK) signaling pathway (Figure 5A, Supplementary Table 1). Additionally, stratified GSEA revealed that some apoptosis-related pathways and biological processes, such as Notch signaling pathway, p53 signaling pathway, and apoptotic DNA fragmentation, were significantly enriched in the high-risk group (Figure 5B, Supplementary Table 2).

### Exploration and Validation of Gene Expression in the Signature

**Figure 6:**
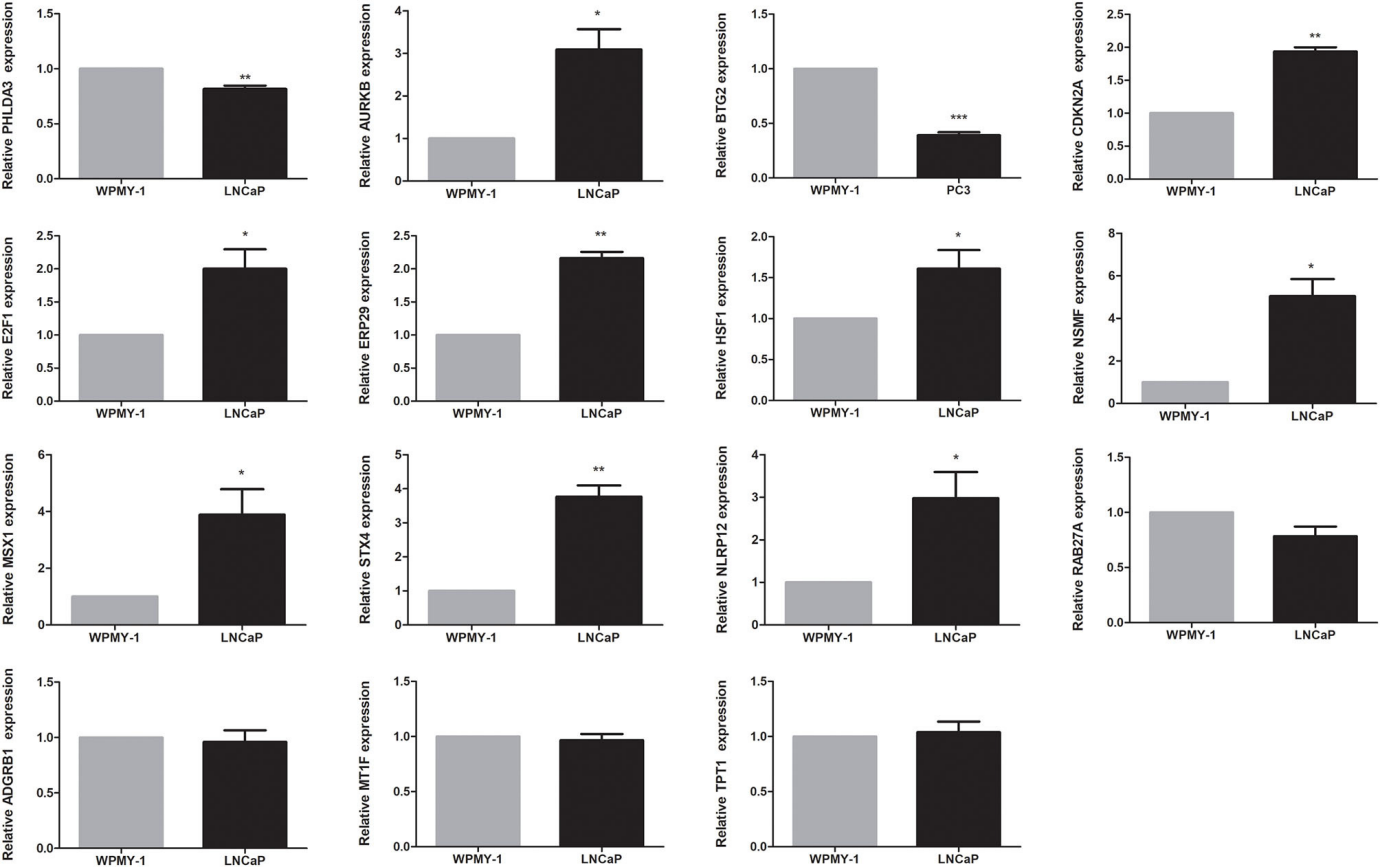
Validation of expression of genes in the signature by quantitative real-time PCR (qRT-PCR). *P < 0.05, **P < 0.01, ***P < 0.001.

The expression levels of genes in the signature in TCGA dataset were shown in Supplementary Figure 3. Among 15 genes in the signature, except four genes, *RAB27A, ADGRB1, MT1F*, and *TPT1*, the remaining were differentially expressed between tumor and normal tissues. Among them, nine genes (*AURKB, CDKN2A, E2F1, ERP29, HSF1, NSMF, MSX1, STX4*, and *NLRP12*) were upregulated, while the other two genes (*PHLDA3* and *BTG2*) were downregulated in the PCa tissues. We found that all 15 genes were significantly associated with BCR. Among them, *PHLDA3, BTG2, ERP29, NLRP12, RAB27A, MT1F*, and *TPT1* were protective genes, whereas *AURKB, CDKN2A, E2F1, HSF1, NSMF, MSX1, STX4*, and *ADGRB1* were risky genes (Supplementary Figure 4). To further determine the expression of these genes, we used immunohistochemistry results from the Human Protein Atlas database to show that *AURKB, CDKN2A, E2F1, ERP29, HSF1, NSMF, MSX1, STX4*, and *NLRP12* were significantly increased in PCa compared with normal tissue. In contrast, the antibody staining levels of *PHLDA3* and *BTG2* were relatively reduced in PCa tissue. Additionally, there were no significant differences in the protein expression of *RAB27A* and *TPT1* between tumor and normal tissues (Supplementary Figure 5). Finally, the results from qRT-PCR validation were also consistent with the bioinformatics results, which further confirmed the accuracy of our bioinformatics analysis (Figure 6).

## Discussion

After RP, PSA will drop to undetectable levels as expected, and BCR is defined as two consecutive serum PSA >0.2 ng/ml and rising (Moul, 2000). BCR is a sign of a major progression and is associated with clinical recurrence, metastasis, and cancer-specific mortality. As reported, 24–34% of patients with BCR will develop metastasis and receive androgen deprivation therapy as recommended in guidelines (Pound et al., 1999; Boorjian et al., 2011). Unfortunately, it is a largely palliative care, as most of them will eventually develop into metastatic castration-resistant PCa (Heinlein and Chang, 2004). The best way to improve these patients' prognosis is to identify high-risk patients at an early stage and adopt more rigorous follow-up plans and personalized adjuvant therapies. Currently, predictions of BCR are mainly based on clinical parameters with a low predictive accuracy. According to the guidelines, patients are divided into low-, medium-, and high-risk groups based on PSA, GS, and cT (Mottet et al., 2017). Patients in the high-risk group receive adjuvant or other systemic therapies, while those with low risk undergo active surveillance. However, the performance of this risk stratification is unsatisfactory, and a few patients in the low-risk group develop BCR (Ghadjar et al., 2018). Thus, it is of great clinical value to find novel biomarkers with more predictive accuracy for BCR in localized PCa after RP.

In this study, we established a 15-gene signature based on apoptosis-related genes in the training set and validated it in the other three independent validation sets. WGCNA was conducted to identify the candidate gene module most correlated with BCR, and univariate and multivariate Cox regression analyses were performed to construct the gene signature with genes from the candidate module. Fifteen genes were finally enrolled in the signature, and each patient's risk score was calculated according to the formula. We found that patients with a high-risk score had a significantly poorer RFS than those with a low-risk score in each cohort. In multivariate Cox regression analysis, after correction for clinical parameters, the risk score still served as an independent prognostic factor for BCR in each cohort. Time-dependent ROC analysis revealed that the risk score had the strongest predictive power in both the training set and the validation sets. It is worth noting that the risk score was the only significant predictor in all four cohorts, with an average AUC ranging from 0.761 to 0.899. Moreover, after *Z*-score normalization, the prognostic value of the risk score was satisfactory in the pooled cohort.

To meet clinical needs and improve risk stratification, we combined the risk score with four clinical variables (GS, PSA, SM, and cT) and constructed a nomogram based on two of the cohorts. The nomogram model enabled practitioners to predict 1-, 3-, and 5-year RFS of localized PCa patients after RP. Time-dependent ROC analysis demonstrated that the prognostic accuracy significantly improved after the risk score was combined with clinical parameters, with an average AUC of 0.855 after a 5-year follow-up. Additionally, the presentation of calibration plot showed that there was good conformity between the predicted and observed outcomes.

Some of the genes in our signature have been reported to be involved in cancer, especially PCa. For example, *BTG2*, a protective gene in the signature, was downregulated in PCa, and the ectopic expression of this gene inhibited PCa cell growth. Hu et al. (2011) discovered that *BTG2* complexes with androgen receptors (ARs) *via* an LxxLL-dependent mechanism and may play a role in PCa by modulating the AR signaling pathway. Nuclear *HSF1* expression could serve as a novel prognostic marker for PCa patient risk stratification for disease progression and survival after RP (Bjork et al., 2018). Worst et al. (2017) reported that *RAB27A* was frequently underexpressed in advanced PCa and was inversely correlated with PCa outcome. In addition to the abovementioned genes, some genes play an important role in the progression and prognosis of other tumors as well, such as *AURKB* in clear cell renal cell carcinoma (Wan et al., 2019) and gastric cancer (Nie et al., 2020), *MSX1* in melanoma (Heppt et al., 2018) and breast cancer (Yue et al., 2018), *ERP29* in osteosarcoma (Chaiyawat et al., 2019), and *MT1F* in gastric cancer (Lin et al., 2017). Taken together, the functional roles of 15 genes in the signature in PCa and their underlying mechanisms still require further research.

Functional enrichment analysis demonstrated that genes in the candidate module were significantly enriched in some apoptosis-related biological processes and pathways. In addition, GSEA revealed that some apoptosis-related pathways and biological processes were significantly enriched in the high-risk group as well. The control of apoptotic mechanisms is integral to many aspects of tumor biology and appears to be involved in the process of recurrence in several malignancies, such as malignant melanoma (Xu et al., 2019), glioma (Lan et al., 2018), and PCa (Hirata et al., 2009; Anees et al., 2011; Khan et al., 2014). Apoptosis serves as an essential mechanism to prevent the proliferation of cells with a higher mutation rate, thus tempering malignant transformation. Resistance to treatment may result from specific inhibition of apoptotic signaling (Konstantinidou et al., 2002). Notably, the p53 signaling pathway was enriched in both analyses. Moreover, some genes in the signature, like *HSF1* (Toma-Jonik et al., 2019) and *E2F1* (Udayakumar et al., 2010), have been reported to be associated with the p53 signaling pathway. Thus, we suppose that the p53 signaling pathway might contribute to cancer progression and recurrence and poor prognosis in PCa.

We acknowledge some limitations of this study. Firstly, due to the retrospective design and relatively small sample size, multicenter and prospective studies are needed to validate the predictive value of our signature. Secondly, some cohorts are with incomplete clinical information, such as age and PSA. Thirdly, in addition to PCR, more in-depth experiments are needed to further explore the biological functions underlying the signature in PCa. However, the prognostic value of our signature for BCR in localized PCa after RP cannot be ignored.

## Conclusion

Taken together, this study identified a novel robust apoptosis-related gene signature for BCR in localized PCa patients after RP. Incorporation of the gene signature into clinical parameters could further improve risk stratification. However, well-designed prospective studies are needed to further validate its prognostic value and test its clinical utility.

## Data Availability Statement

Publicly available datasets were analyzed in this study. This data can be found here: https://portal.gdc.cancer.gov.

## Author Contributions

QZ, JX and NS designed the manuscript. QZ, CJ, RC, JL, and XZ completed the data download and analysis. QZ, KZ, and LS wrote the manuscript. All the authors approved the final manuscript.

## Conflict of Interest

The authors declare that the research was conducted in the absence of any commercial or financial relationships that could be construed as a potential conflict of interest.

## Supplementary Material

Supplementary Figure 1 WGCNA analysis in PCa. **(A)** Sample clustering was performed to exclude outliers; **(B)** Analysis of the scale-free fit index for various soft-thresholding powers (left) and the mean connectivity for various soft-thresholding powers (right); **(C)** Correlation between apoptosis-related genes and BCR in other modules. PCa, prostate cancer; BCR, biochemical recurrence.

Supplementary Figure 2 A predictive nomogram based on clinical variables. **(A)** Nomogram plot to predict 1-, 3-, and 5-year survival; **(B)** ROC curves of the nomogram to predict 1-, 3-, and 5-year survival.

Supplementary Figure 3 Expression level of genes in the signature in TCGA dataset.

Supplementary Figure 4 Kaplan-Meier survival curves of all genes in the signature in TCGA cohort.

Supplementary Figure 5 Validation of protein expression of genes in the signature in normal tissue and PCa using the HPA database.

Supplementary Table 1 GO and KEGG about genes in candidate module related to BCR.

Supplementary Table 2 Gene set enrichment analyses between high- and low-risk group in the training cohort (terms enriched in high-risk group).
