# Supplementary material for: A Novel Apoptosis-Related Gene Signature Predicts Biochemical Recurrence of Localized Prostate Cancer After Radical Prostatectomy
Source: Front Genet. 2020 Nov 30;11:586376. doi: 10.3389/fgene.2020.586376 (PMC7734189; doi:10.3389/fgene.2020.586376)

Supplementary table1. GO and KEGG about genes in candidate module related to BCR

| Items | | -logP |
| --- | --- | --- |
| GO | |  |
| BP | regulation of apoptosis | 26.92 |
|  | regulation of programmed cell death | 26.72 |
|  | regulation of cell death | 26.64 |
|  | negative regulation of apoptosis | 15.08 |
|  | positive regulation of apoptosis | 15.05 |
| CC | extracellular space | 5.85 |
|  | extracellular region part | 4.28 |
|  | cytoplasmic membrane-bounded vesicle | 2.89 |
|  | cytoplasmic vesicle | 2.80 |
|  | membrane-bounded vesicle | 2.77 |
| MF | identical protein binding | 3.85 |
|  | eukaryotic cell surface binding | 3.39 |
|  | protein dimerization activity | 3.04 |
|  | kinase binding | 3.00 |
|  | protein kinase binding | 2.72 |
| KEGG | |  |
|  | Pathways in cancer | 2.51 |
|  | p53 signaling pathway | 2.17 |
|  | Huntington's disease | 1.82 |
|  | Amyotrophic lateral sclerosis (ALS) | 1.68 |
|  | MAPK signaling pathway | 1.55 |

Supplementary table2. Gene set enrichment analyses between high- and low-risk group in the training cohort (terms enriched in high-risk group)

| Items | Size | NES | P-value |
| --- | --- | --- | --- |
| C2 KEGG | | | |
| KEGG_BASE_EXCISION_REPAIR | 33 | 1.98 | 0.014 |
| KEGG_SNARE_INTERACTIONS_IN_VESICULAR_TRANSPORT | 38 | 1.96 | 0.004 |
| KEGG_CELL_CYCLE | 124 | 1.94 | 0.016 |
| KEGG_OOCYTE_MEIOSIS | 112 | 1.93 | 0.006 |
| KEGG_SPLICEOSOME | 126 | 1.86 | 0.023 |
| KEGG_NOTCH_SIGNALING_PATHWAY | 47 | 1.57 | 0.036 |
| KEGG_P53_SIGNALING_PATHWAY | 68 | 1.51 | 0.042 |
| C5 BP | | | |
| GO_SPLICEOSOMAL_COMPLEX_ASSEMBLY | 56 | 2.05 | 0.008 |
| GO_EXIT_FROM_MITOSIS | 31 | 2.05 | 0.002 |
| GO_CELLULAR_COMPONENT_DISASSEMBLY_INVOLVED_IN_EXECUTION_PHASE_OF_APOPTOSIS | 35 | 1.98 | 0.002 |
| GO_METAPHASE_ANAPHASE_TRANSITION_OF_CELL_CYCLE | 56 | 1.82 | 0.036 |
| GO_REGULATION_OF_CELL_CYCLE_G2_M_PHASE_TRANSITION | 211 | 1.82 | 0.023 |
| GO_APOPTOTIC_DNA_FRAGMENTATION | 24 | 1.82 | 0.012 |
| GO_NEGATIVE_REGULATION_OF_METAPHASE_ANAPHASE_TRANSITION_OF_CELL_CYCLE | 37 | 1.81 | 0.030 |
| GO_CELL_CYCLE_G2_M_PHASE_TRANSITION | 265 | 1.81 | 0.027 |
| GO_REGULATION_OF_CYSTEINE_TYPE_ENDOPEPTIDASE_ACTIVITY_INVOLVED_IN_APOPTOTIC_SIGNALING_PATHWAY | 17 | 1.77 | 0.012 |
| GO_REGULATION_OF_CELL_CYCLE_PHASE_TRANSITION | 434 | 1.75 | 0.027 |
| C5 CC | | | |
| GO_NUCLEAR_UBIQUITIN_LIGASE_COMPLEX | 43 | 1.99 | 0.004 |
| GO_ANAPHASE_PROMOTING_COMPLEX | 21 | 1.93 | 0.008 |
| GO_MICROTUBULE_ORGANIZING_CENTER_PART | 177 | 1.88 | 0.001 |
| GO_SPLICEOSOMAL_COMPLEX | 174 | 1.88 | 0.018 |
| GO_CENTRIOLE | 133 | 1.87 | 0.002 |
| GO_CENTRIOLAR_SATELLITE | 29 | 1.85 | 0.004 |
| GO_H4_H2A_HISTONE_ACETYLTRANSFERASE_COMPLEX | 18 | 1.85 | 0.004 |
| C5 MF | | | |
| GO_SNAP_RECEPTOR_ACTIVITY | 31 | 1.86 | 0.006 |
| GO_CYCLIN_DEPENDENT_PROTEIN_SERINE_THREONINE_KINASE_REGULATOR_ACTIVITY | 47 | 1.82 | 0.006 |
| GO_ACID_AMINO_ACID_LIGASE_ACTIVITY | 15 | 1.76 | 0.010 |
| GO_DNA_POLYMERASE_BINDING | 17 | 1.64 | 0.048 |
| GO_UBIQUITIN_PROTEIN_TRANSFERASE_REGULATOR_ACTIVITY | 17 | 1.64 | 0.042 |
| GO_CATALYTIC_ACTIVITY_ACTING_ON_DNA | 177 | 1.73 | 0.045 |
| GO_DEOXYRIBONUCLEASE_ACTIVITY | 57 | 1.61 | 0.041 |
| GO_H4_HISTONE_ACETYLTRANSFERASE_ACTIVITY | 21 | 1.61 | 0.026 |

Supplementary figure1.


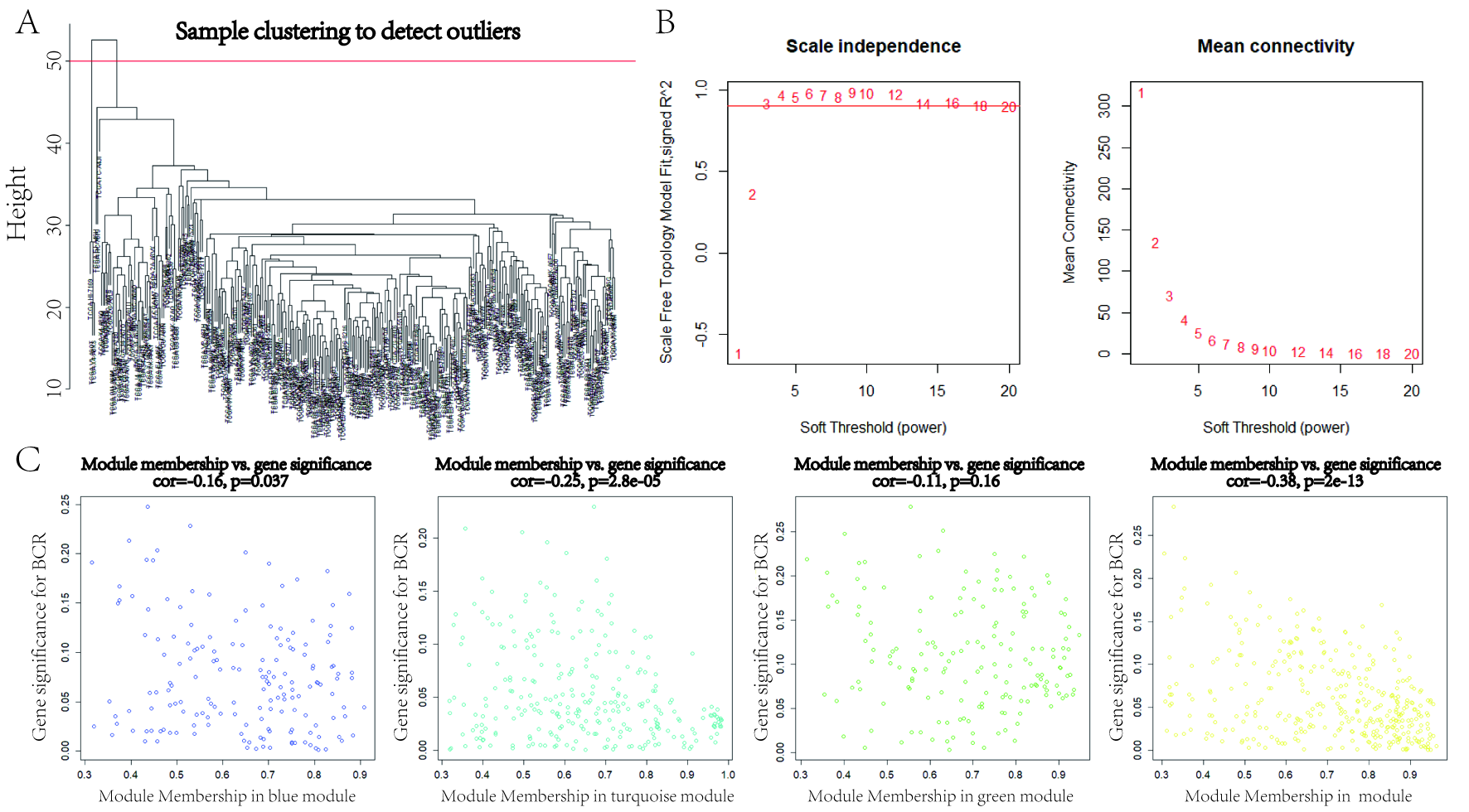


Supplementary figure2.


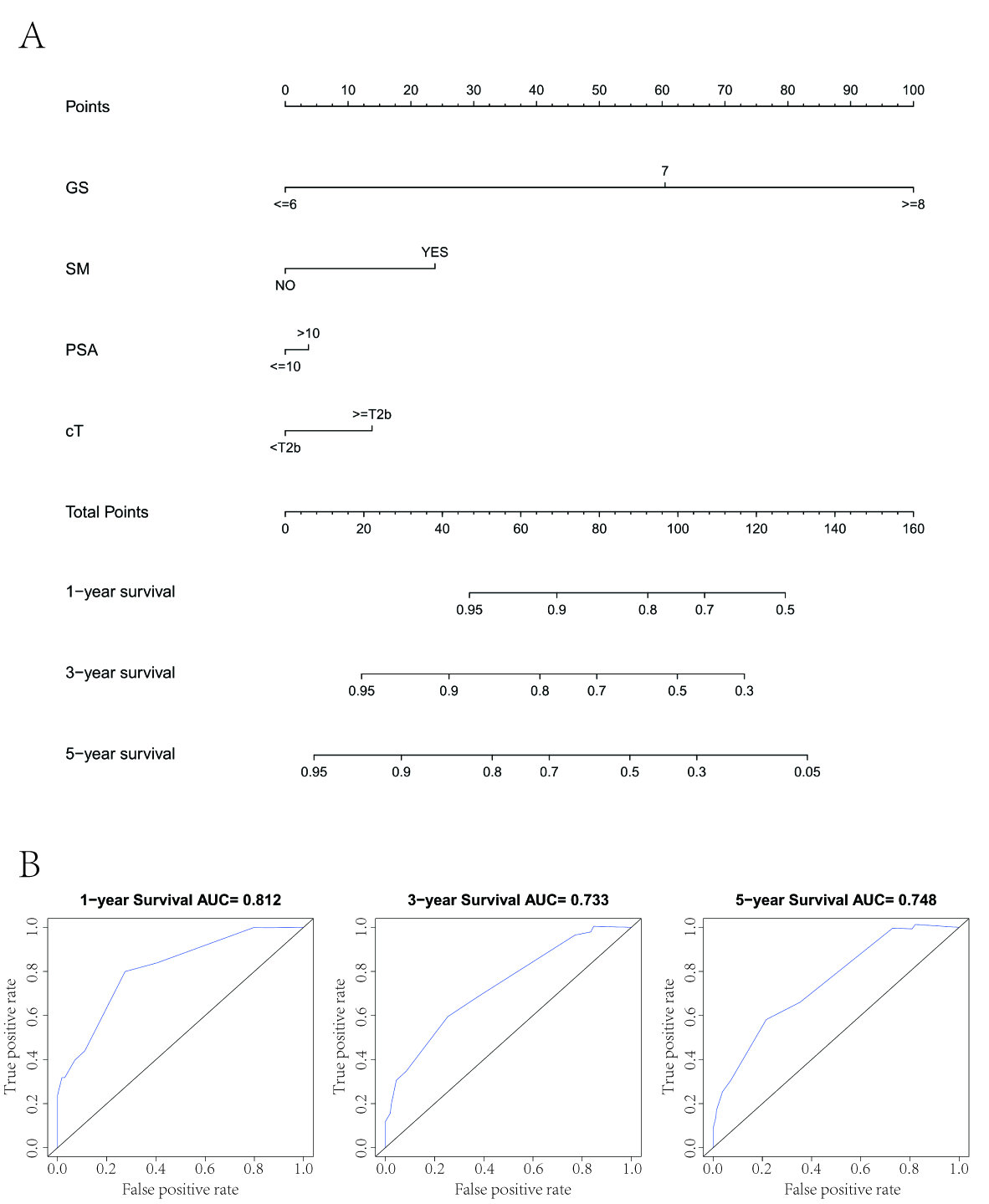


Supplementary figure3.


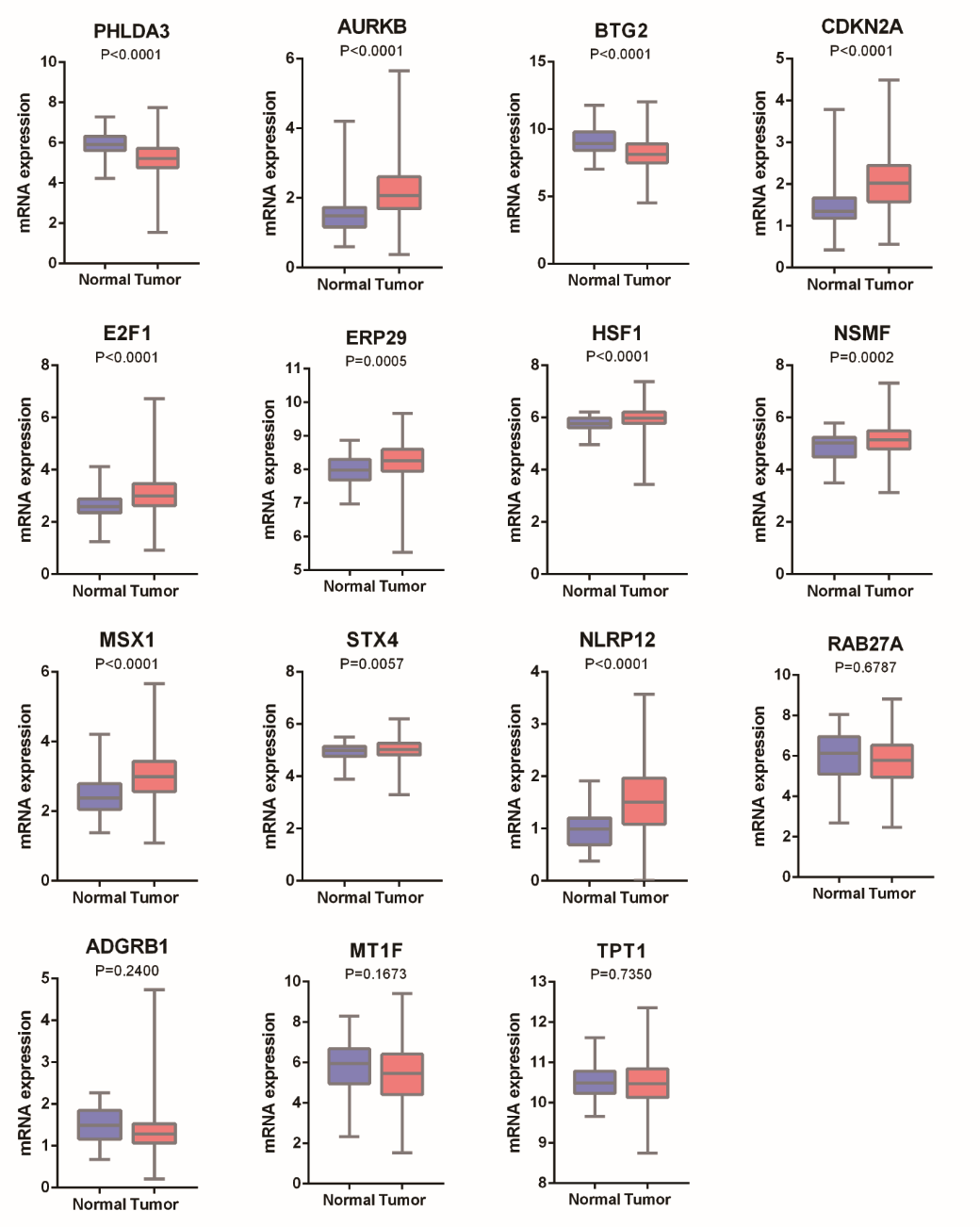


Supplementary figure4.


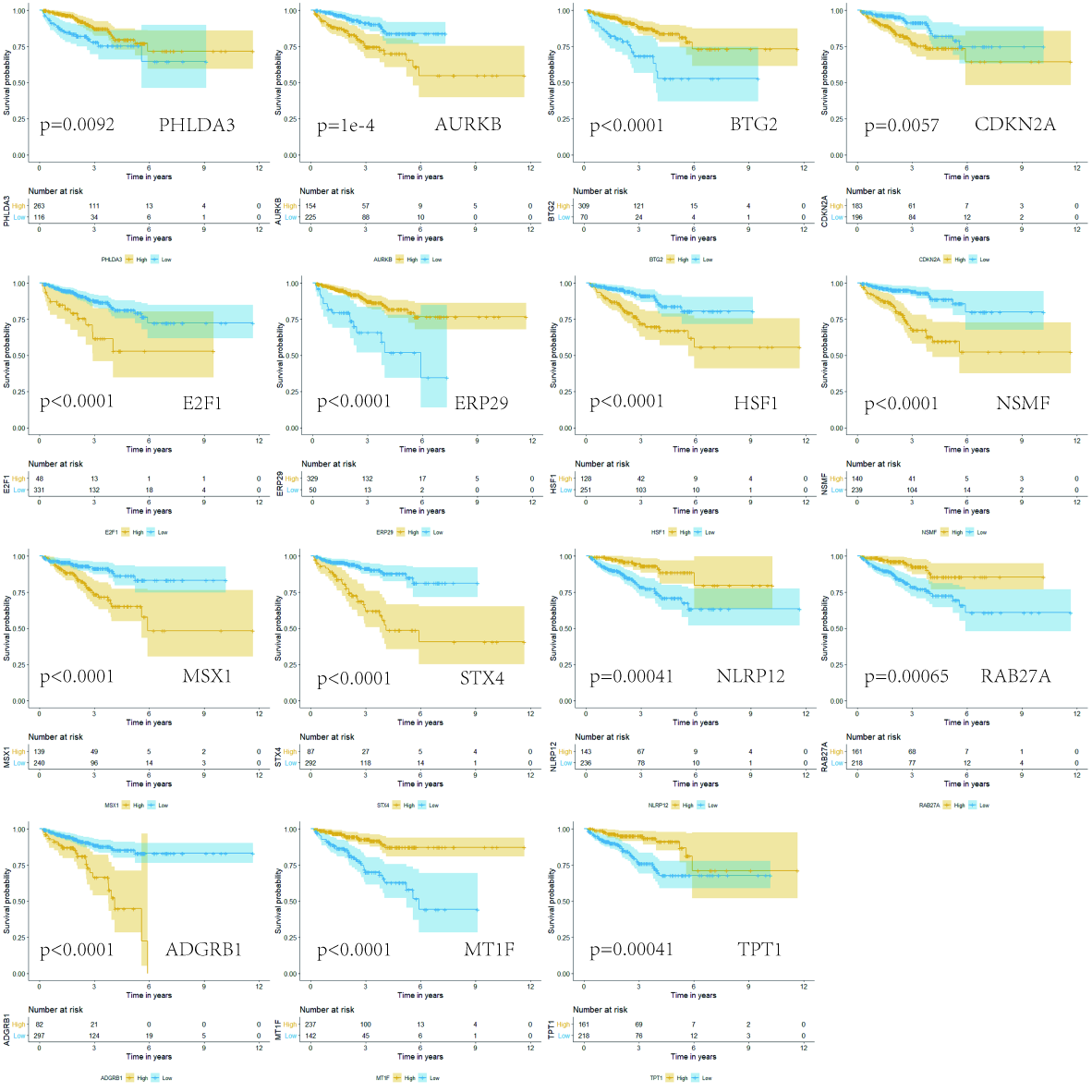


Supplementary figure5.


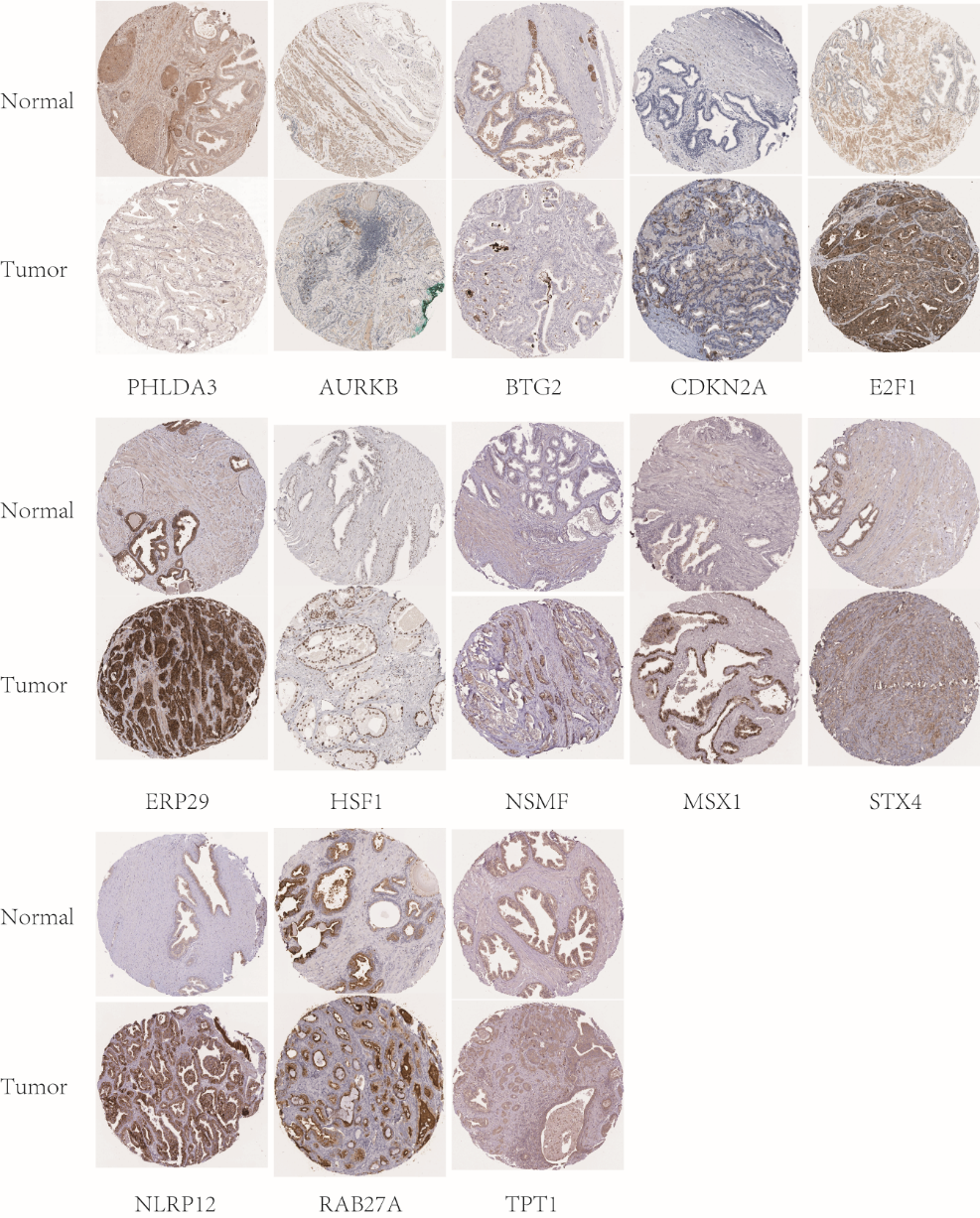

Supplement: Supplementary Figure 1 — WGCNA analysis in PCa. (A) Sample clustering was performed to exclude outliers; (B) Analysis of the scale-free fit index for various soft-thresholding powers (left) and the mean connectivity for various soft-thresholding powers (right); (C) Correlation between apoptosis-related genes and BCR in other modules. PCa, prostate cancer; BCR, biochemical recurrence. [file Data_Sheet_1.docx]
